# Short peptide perturbs spermatogenesis via immune microenvironment dysregulation and mitochondrial imbalance

**DOI:** 10.1002/2211-5463.70058

**Published:** 2025-05-22

**Authors:** Heng Wang, Xiaofang Tan, Deyu Chen

**Affiliations:** ^1^ School of Basic Medical Science Guangzhou Medical University China; ^2^ Affiliated Maternity and Child Health Care Hospital of Nantong University China; ^3^ College of Medicine Fuyang Normal University China

**Keywords:** apoptosis, blood–testis barrier, inflammation, mitophagy, spermatogenesis, tight junctions

## Abstract

A short peptide derived from the occludin protein regulates tight junctions (TJ) of the blood–testis barrier and impairs germ cell development. However, the mechanism behind how this peptide regulates TJ and induces cell apoptosis remains unclear. In the present study, an animal model with induced TJ disruption via the short peptide was used to evaluate its impact on spermatogenesis. Here, we demonstrate that the short peptide promoted the infiltration of immune cells into the testicular interstitial tissue, accompanied by upregulation expression of the pro‐inflammatory factors interleukin‐6 and tumor necrosis factor‐α. Moreover, mitochondrial fragmentation and mitophagy were upregulated in Sertoli cells and Leydig cells. Consistently, terminal deoxynucleotidyl transferase dUTP nick end labeling staining revealed extensive apoptosis in the testes during spermatogenesis. Notably, the severity of these disruptions began to attenuate after 27 days, although full functional recovery was not observed. Our findings reveal a novel mechanism wherein peptide‐induced immune dysregulation and mitochondrial dysfunction synergistically impair spermatogenesis, potentially via microenvironmental perturbation of the TJ. Overall, these findings could hold valuable insights for the development of non‐hormonal male contraceptives.

## Graphical abstract

In the blood–testis barrier, occludin is crucial for tight junctions. This study demonstrates that occludin‐targeting short peptides disrupt junction integrity, inducing immune cell infiltration, tumor necrosis factor‐α/interleukin‐6 secretion and mitochondrial dysfunction, ultimately triggering apoptosis. Notably, these pathological manifestations showed significant attenuation after 27 days after intervention, suggesting time‐dependent recovery of barrier function after peptide‐induced disruption.

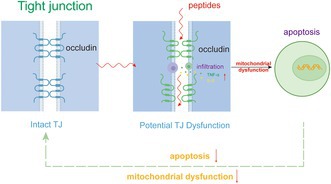

**Abbreviations**
AA: amino acids
BTB: blood–testis barrier
DRP1: dynamin‐related protein 1
IL: interleukin
MMP: matrix metalloproteinase
MFN1: mitofusin 1
PBS: phosphate‐buffered saline
RT‐qPCR: reverse transcription quantitative PCR
TJ: tight junction
TNF: tumor necrosis factor
Treg: regulatory T cell
TUNEL: terminal deoxynucleotidyl transferase dUTP nick end labeling

In the testes, spermatogonial stem cells give rise to generate spermatogonia, which subsequently differentiate into spermatocytes, sperm cells and ultimately mature sperm [1]. Spermatogenesis is a complex biological process. The blood–testis barrier (BTB) is a critical testicular structure essential for spermatogenesis [2]. The BTB forms a barrier in the seminiferous tubules, preventing harmful substances in the blood from infiltrating and causing sperm damage. Additionally, it prevents the immune system in the testes from recognizing germ cells as foreign antigens and attacking them [3]. Its integrity establishes and maintains a favorable microenvironment for sperm production.

In mammalian testes, the BTB is primarily composed of adhesive junctions desmosomes, gap junctions and tight junctions (TJs) between Sertoli cells [4, 5]. Proper formation and opening of TJs establish a stable microenvironment conducive to sperm formation within the BTB. The TJs of the BTB consist of various TJ proteins, including occludin, the claudin family and the junction adhesion molecule family [6]. Disruptions of multiple TJ proteins impair the function of the BTB. Disorganization of claudin‐3, ‐5 and ‐11, as well as occludin, within the BTB impairs its function, leading to the impairment of spermatogenic function [7, 8].

Certain members of the matrix metalloproteinase (MMP) family have been identified to degrade TJ proteins such as occludin, claudin and zonula occludens‐1 [9, 10, 11, 12, 13]. For example, MMP‐8 can degrade occludin in SC cells, leading to the disassembly of cell junction components and male reproductive disorders [9]. It has also been identified that MMP‐7 impairs the intestinal epithelial barrier by cleaving claudin‐7 in inflammatory bowel diseases [11]. Occludin was the first transmembrane TJ protein to be identified that spans the membrane four times to form two extracellular loops and two intracellular loops [14, 15]. It has been reported that multiple extracellular and transmembrane domains of occludin are implicated in the regulation of the TJ barrier [16]. Using an occludin extracellular loop peptide blocks the extracellular loop, disrupting TJ and altering cell membrane permeability [17, 18]. Our previous research reported that a 22 amino acids (AA) short peptide in the second extracellular loop of occludin regulates TJ and cell apoptosis [19]. However, the mechanism by which the 22AA short peptide regulates TJ and induces cell apoptosis remains poorly understood. To further investigate the mechanisms by which the 22AA short peptide disrupts the TJs of the BTB and induces cell apoptosis, the present study analyzed its effects with respect to inducing immune infiltration, promoting mitochondrial autophagy and inducing cell apoptosis.

## Materials and methods

### Animals and treatment

Specific pathogen‐free grade adult healthy KM mice, aged 6–8 weeks old and weighing approximately 25 g, were provided by Chongqing Enswell Biotechnology Co., Ltd (Chongqing, China). The animal facility operates under a 12:12 h light/dark photocycle, with temperatures regulated in the range 23–25 °C, ensuring that animals have *ad libitum* access to food and water. The experiment was approved by the Experimental Animal Ethics Committee of Fuyang Normal University, China (Grant No. 20200006). At the experiment reached endpoints, all mice were euthanized with carbon dioxide and cervical dislocation. Animal models of short peptide‐induced TJ disruption, as described in a previous study [19], were established to investigate the mechanism by which the short peptide inhibits spermatogenesis. Mice were injected intratesticularly with 20 μL of the blocking peptide solution [4.0 mg·mL^−1^ in phosphate‐buffered saline (PBS)]. Control mice received an equal volume of PBS. Procedures were performed under anesthesia using 7% chloral hydrate (0.5 mL per 100 g body weight) in specific pathogen‐free conditions.

### Isolation and culture of primary Sertoli cells

Sertoli cells separation was performed according to previous reports. Briefly, under sterile conditions, bilateral testicles were taken from the abdomen of mice, and 0.125% trypsin was added and digested at a rate of 1.5 mL per testicle for 20 min. Fresh Dulbecco's modified Eagle's medium‐F12 medium supplemented with 10% FBS was used until clusters of Sertoli cells and spermatogenic cells were observed under a microscope. The medium was changed every 3–4 days. Sertoli cells were treated with the peptide at a final concentration of 4 μm (or PBS as control) for different points in serum‐free Dulbecco's modified Eagle's medium/F12 medium.

### Reverse transcription quantitative PCR (RT‐qPCR) analysis

Testicular tissue and Sertoli cells were collected at different time points and total RNA was extracted and reverse‐transcribed into cDNA. Each cDNA sample was amplified using the SYBR Green (Takara, Kusatsu, Japan) with the ABI StepOnePlus PCR Detection System (Thermo Fisher Scientific, Waltham, MA, USA). The primers used are listed in Table 1.

**Table 1:** RT‐qPCR primers sequences used in the present study.

Gene	Sequence (5′‐ to 3′)
TNF‐α‐F	GGCGGTGCCTATGTCTCAG
TNF‐α‐R	TCTCCTCCACTTGGTGGTTTGT
IL‐6‐F	AAGAGACTTCCATCCAGTTGCC
IL‐6‐R	AATTGCCATTGCACAACTCTTT
Mfn1‐F	AAAGCCATCACTGCAATCTTCG
Mfn1‐R	TTGTCCTGCCAAAAAATGCC
Drp1‐F	ATTCCATTATCCTCGCCGTCA
Drp1‐R	AACCCTTCCCATCAATACATCC
Pink 1‐F	GATCCAGGCAATTTTTACACAGA
Pink 1‐R	GGGCATGGTGGCTTCATACA
Parkin‐F	CTCAGACAAGGACACGTCGGTAG
Parkin‐R	GTGACGGTGGTTACACTGGAAGA
Mus‐GAPDH‐F	CAGAAGGGGCGGAGATGAT
Mus‐GAPDH‐R	AGGCCGGTGCTGAGTATGTC

### Detection of mitophagy‐related proteins via western blot

Testicular tissue was cut into small fragments, lysed with RIPA buffer and total protein was extracted. The protein concentration was determined using a BCA assay kit (Thermo Fisher Scientific), and each sample was loaded with 20 μg of protein. After separating the proteins using SDS/PAGE at a voltage of 80–120 V, the samples were transferred to a poly(vinylidene difluoride) membrane. The poly(vinylidene difluoride) membrane was blocked with skim milk powder for 1 h and then incubated overnight with anti‐PINK (monoclonal antibody, ab216144; Abcam, Cambridge, UK) and anti‐Parkin antibodies (monoclonal antibody, ab77924; Abcam). Electrophoretic bands were visualized using the Tanon‐4200 gel imaging system (Tanon Science & Technology Co., Ltd, Shanghai, China). GAPDH (monoclonal antibody ab181602; Abcam) was used as the internal control, and the expression levels of PINK and Parkin were determined by the ratio of their gray values to that of GAPDH.

### Immunofluorescence analysis

Paraffin‐embedded sections were deparaffinized in xylene, followed by a series of ethanol washes (100%, 95%, 85%, 75%), and then rehydrated. Antigen retrieval was performed by sub‐boiling the sections in EDTA buffer (pH 8) at 95–98 °C for 10 min, followed by quenching of endogenous peroxidase activity with 0.3% H_2_O_2_ for 10 min. The sections were subsequently blocked with sheep serum, followed by overnight incubation with the primary antibody at 4 °C. Subsequently, all sections were incubated with a fluorescent secondary antibody for 30 min. Next, the sections were incubated with 4′,6‐diamidino‐2‐phenylindole in the dark for 5 min for nuclear staining. For triple immunofluorescence staining, tissue sections were incubated with the following primary antibodies: (A) mouse monoclonal anti‐CD29 (dilution 1 : 100, #MA1‐06906; Invitrogen, Waltham, MA, USA) for 1 h, (B) rabbit polyclonal anti‐CD4 (dilution 1 :100, #PA5‐87425; Invitrogen) for 1 h and (C) rat monoclonal anti‐CD68 (dilution 1 : 100, ab53444; Abcam) for 1 h. Between each incubation step, sections were washed with PBS (3 × 5 min). Species‐specific secondary antibodies from Abcam were subsequently applied: goat anti‐mouse IgG‐Alexa Fluor 488 (dilution 1 : 1000, #ab150113), goat anti‐rabbit IgG‐Cy3 (dilution 1 : 1000, #ab6939) and goat anti‐rat IgG‐Cy5 (dilution 1 : 1000, #ab6565) for 1 h at 37 °C in the dark.

### Determination of apoptotic cells via the terminal deoxynucleotidyl transferase dUTP nick end labeling (TUNEL) assay

Paraffin‐embedded sections were deparaffinized in xylene, followed by a series of ethanol washes (100%, 95%, 85%, 75%), and then rehydrated. The sections were treated with trypsin at 37 °C for 15 min, followed by boiling in 0.1 m sodium citrate buffer in a microwave for 5 min. Sertoli cells cultured on coverslips were fixed with 4% paraformaldehyde for 15 min and then permeabilized with 0.1% Triton X‐100 for 2 min. Next, 50 μL of TUNEL reaction mixture was added to the sample and incubated in a 37 °C constant temperature incubator for 60 min. Then, 50 μL of conversion POD was added to the sample and incubated in a constant temperature incubator at 37 °C for 30 min. Following 3,3′‐diaminobenzidine staining and hematoxylin staining, images were observed and captured under a microscope.

### Statistical analysis

The experimental results are presented as the mean ± SD. Data processing was performed using prism, version 9 (GraphPad Software Inc., San Diego, CA, USA). Differences between the control group and experimental group were analyzed using a *t*‐test. Differences among the various experimental groups were examined using one‐way analysis of variance. *P* < 0.05 was considered statistically significant.

## Results

### Short peptide promotes immune cell infiltration

**Fig. 1:**
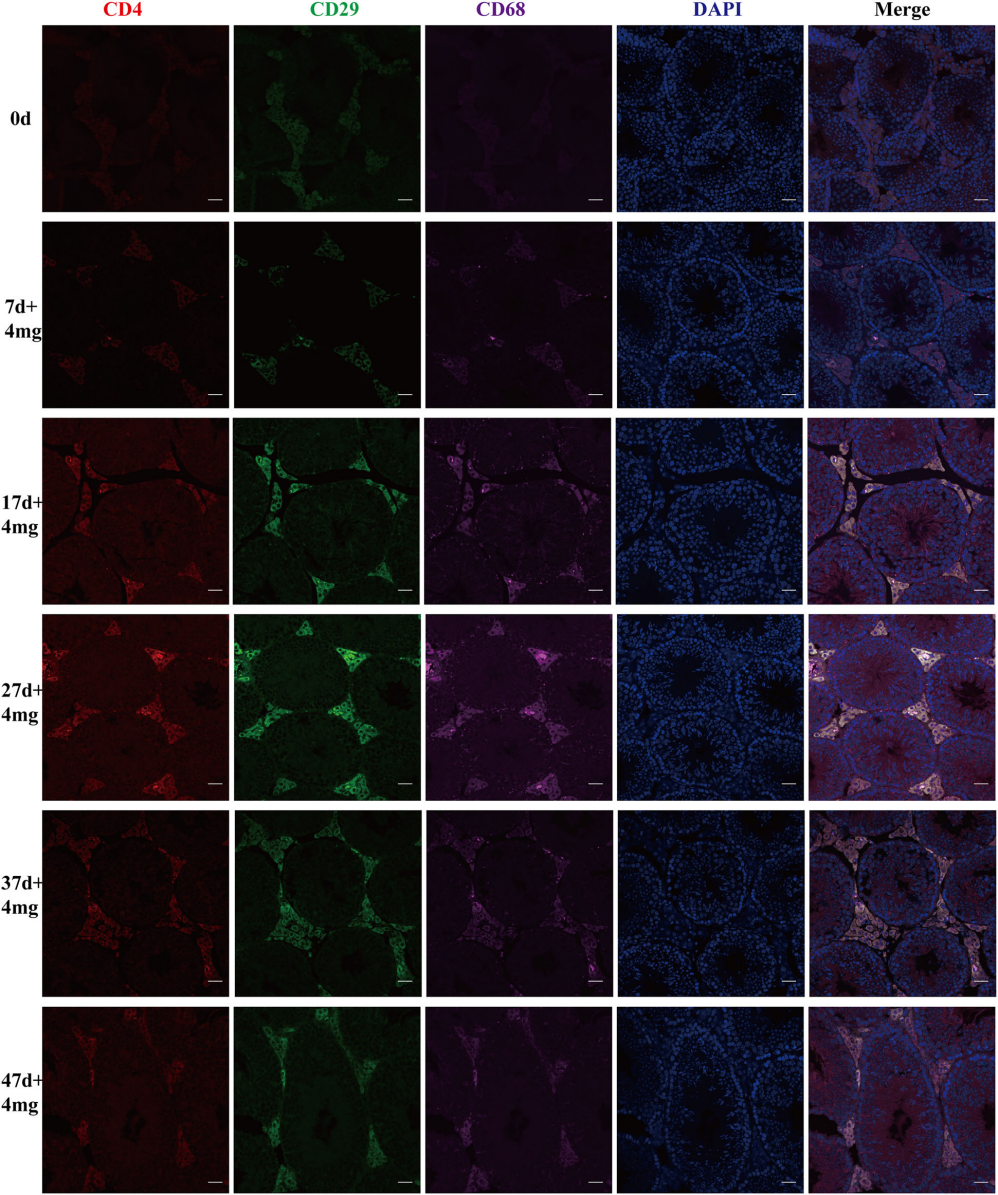
Effect of short peptide on the immune cell infiltration. Multiple immunofluorescence assays were used to detect the infiltration of immune cells. Day 0 was the control group. Experimental group samples were collected at 7 days (+4 mg), 17 days (+4 mg), 27 days (+4 mg), 37 days (+4 mg) and 47 days (+4 mg) after the administration of 4 mg short peptide. Scale bar = 20 μm.

In the testes, the BTB separates the seminiferous tubules from the interstitial tissue, preventing immune cells in the blood from attacking spermatogenic cells [6, 20]. Multiple immunofluorescence assays were employed to detect the infiltration of immune cells in the seminiferous tubules induced by short peptides. As shown in Fig. 1 and Fig. S1, after 14 days of short peptide injection, the infiltration levels of T cells and macrophages in the interstitial tissue were significantly enhanced, reaching a peak at 27 days. However, 37 days later, the infiltration of immune cells weakened, indicating the initiation of TJ restoration to prevent immune cell infiltration. The results indicate that short peptide induces immune cell accumulation in the testicular interstitium. Combined with our prior observation of occludin degradation [19], this suggests a potential association between peptide treatment and TJ disruption.

### Upregulation of pro‐inflammatory factor expression by a short Peptide

**Fig. 2:**
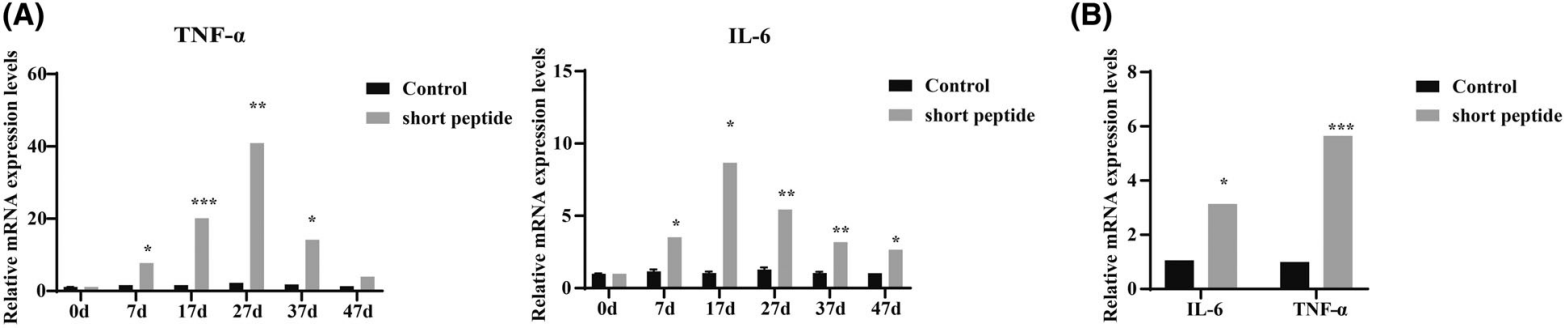
Expression of IL‐6 and TNF‐α mRNA in testes and Sertoli cells. (A) RT‐qPCR was used to detect IL‐6 and TNF‐α mRNA expression in testicular tissue from six different groups. *P < 0.05, **P < 0.01 and ***P < 0.001 vs. the control group (*n* = 3, mean ± SEM). (B) RT‐qPCR was used to detect IL‐6 and TNF‐α mRNA expression in Sertoli cells after 7 days with and without short peptides group (*n* = 3). *P < 0.05 and ***P < 0.001 vs. the control group (*n* = 3, mean ± SEM).

The production of cytokines is a significant function of immune cells in exerting cytotoxic effects. Interleukin (IL)‐6 and tumor necrosis factor (TNF)‐α have been reported to exert different effects on the testicular barrier [21, 22]. Subsequent analysis of the expression levels of inflammatory factors in testicular tissue was conducted (Fig. 2A). The mRNA expression levels of IL‐6 and TNF‐α began to increase significantly on day 7 following short peptide administration, reaching peak levels at 17 and 27, respectively, followed by a decline and reaching lower levels by day 47. This further confirmed that, following short peptide administration, the TJ in the testes begins to break down, allowing immune cells to infiltrate the testicular interstitial tissue, thereby releasing pro‐inflammatory factors.

However, Sertoli cells were found to be an important source of IL‐6 in the testis, with high levels observed during the early stages of *Ureaplasma urealyticum* infection [23]. TNF‐α was upregulated by arecoline in testis cells and mouse testicular Sertoli cell line [24]. Furthermore, Sertoli cells isolated from mouse testes showed upregulation of IL‐6 and TNF‐α mRNA levels in the 4 μm peptide groups compared to the control groups (Fig. 2B).

### Short peptide promotes mitophagy and mitochondrial fragmentation

**Fig. 3:**
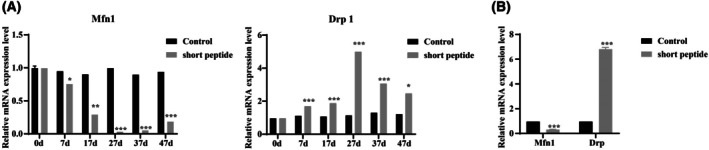
Expression of *Mfn1* and *Drp1* mRNA in testes and Sertoli cells. (A) RT‐qPCR was used to detect *Mfn1* and *Drp1* mRNA expression in testicular tissue from six different groups. *P < 0.05, **P < 0.01 and ***P < 0.001 vs. control (*n* = 3, mean ± SEM). (B) RT‐qPCR was used to detect *Mfn1* and *Drp1* mRNA expression in Sertoli cells after 7 days with and without short peptides group. ***P < 0.001 vs. control group (*n* = 3, mean ± SEM).

Mitochondria are extremely important in regulating the physiological aspects of reproductive function, from spermatogenesis to fertilization [25]. There are two types of mitochondrial dynamics: fusion and fission. Mitofusin 1 (MFN1) is the principal factor influencing mitochondrial fusion. As shown in Fig. 3A, we initially detected the mRNA levels of the mitochondrial fusion gene *Mfn1* in the testis, which began to decline from 7 days, reached its lowest point at 27 days, and started to recover after 37 days, indicating that mitochondrial fusion decreased. By contrast, dynamin‐related protein 1 (DRP1) is the key mediator of mitochondrial fission. The mRNA expression levels of the mitochondrial fission gene *Drp1* began to increase after 7 days, peaked at 27 days, and then gradually recovered after 37 and 47 days, indicating that the small peptides promote mitochondrial fission in cells (Fig. 3A). Meanwhile, in Sertoli cells treated with 4 μm short peptide groups, the mRNA levels of *Mfn1* were found to be downregulated, whereas those of *Drp1* were upregulated (Fig. 3B). These results suggest that the short peptides promote mitochondrial fragmentation.

**Fig. 4:**
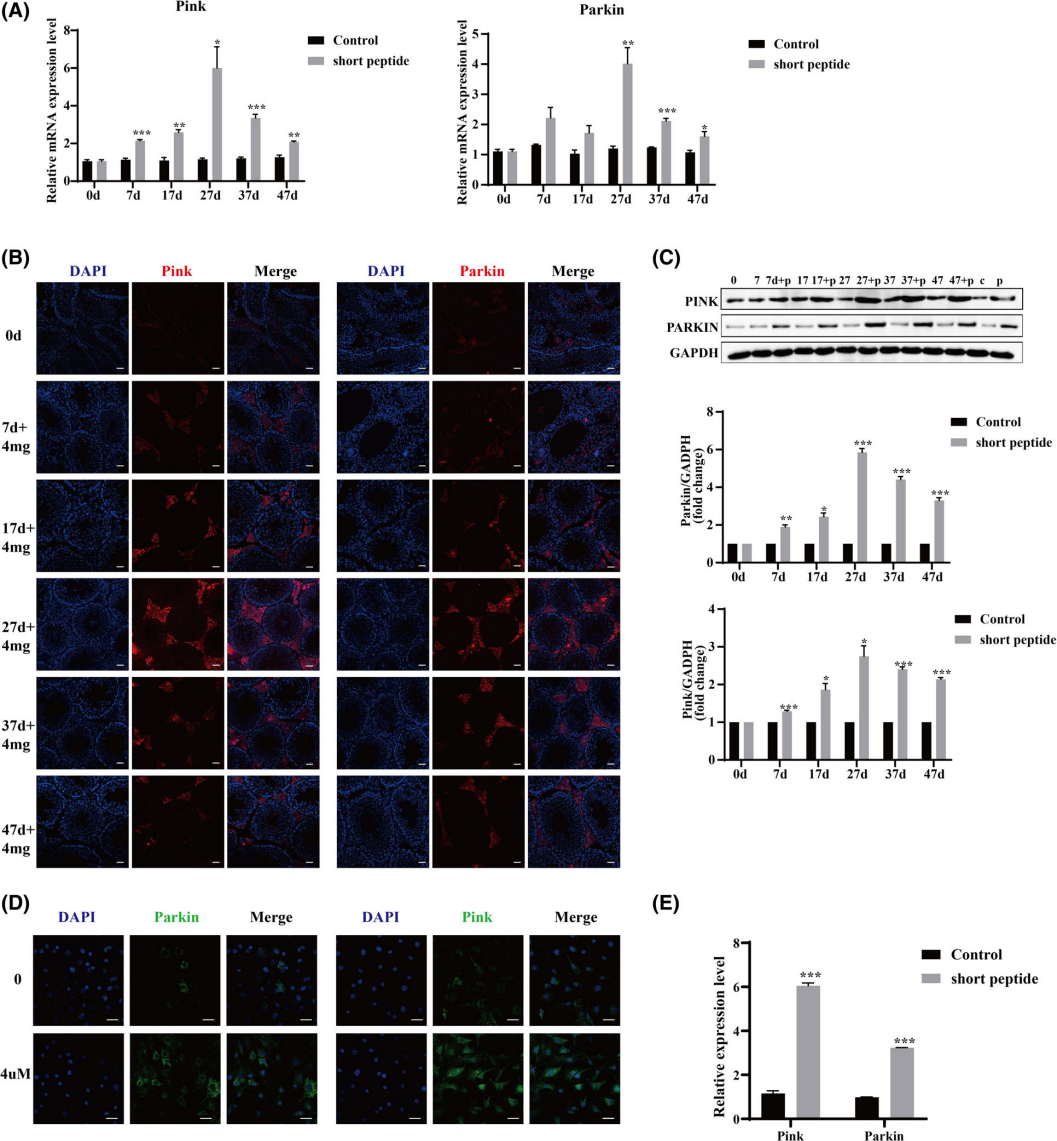
Short peptide attenuated mitochondrial autophagy in testicular tissues and Sertoli cells. RT‐qPCR (A) and western blot (C) were used to detect *Pink* and *Parkin* mRNA expression in testicular tissue from six different groups. (B) The effects of short peptide on *Pink* and *Parkin* expression and localization were analyzed by immunofluorescence in testis. Scale bar = 20 μm. Immunofluorescence (D) and RT‐qPCR (E) were used to detect *Pink* and *Parkin* expression in Sertoli cells. Scale bar = 20 μm. The data represent the mean ± SEM of three independent experiments (*P < 0.05, **P < 0.01 and ***P < 0.001 vs. control; *n* = 3, mean ± SEM).

Next, we examined the expression levels of mitochondrial autophagy‐related genes *Pink1* and *Parkin*. Initially, both mRNA and protein levels of *Pink1* and *Parkin* were increased at 7 and 27 days, with the highest at 27 days (Fig. 4A,C and Figs S2 and S3). Then, the levels decreased at 37 and 47 days, indicating a recovery of autophagy levels. Additionally, we used fluorescence confocal microscopy to further observe the localization changes of *Pink1* and *Parkin*. The results showed that the changes in expression levels were consistent with qunatitative PCR and western blotting (Fig. 4B). Both mitochondrial autophagy genes were initially observed to be upregulated in the testicular interstitium at 7 days, with changes in *Pink1* and *Parkin* observed in the Sertoli cells at 27 days, indicating that the short peptides increase mitochondrial autophagy not only in testicular Leydig cells, but also in some Sertoli cells and spermatogenic cells. This phenomenon began to decrease after 47 days. Additionally, the expression of *Pink1* and *Parkin* was significantly upregulated in Sertoli cells treated with 4 μm short peptide groups (Fig. 4C,E). Immunofluorescence also observed an increase in *Pink1* and *Parkin*‐positive cells in Sertoli cells (Fig. 4D). These results indicate that the short peptide promotes mitochondrial mitophagy in testicular tissues and Sertoli cells.

### Short peptide influences apoptosis to inhibit spermatogenesis

**Fig. 5:**
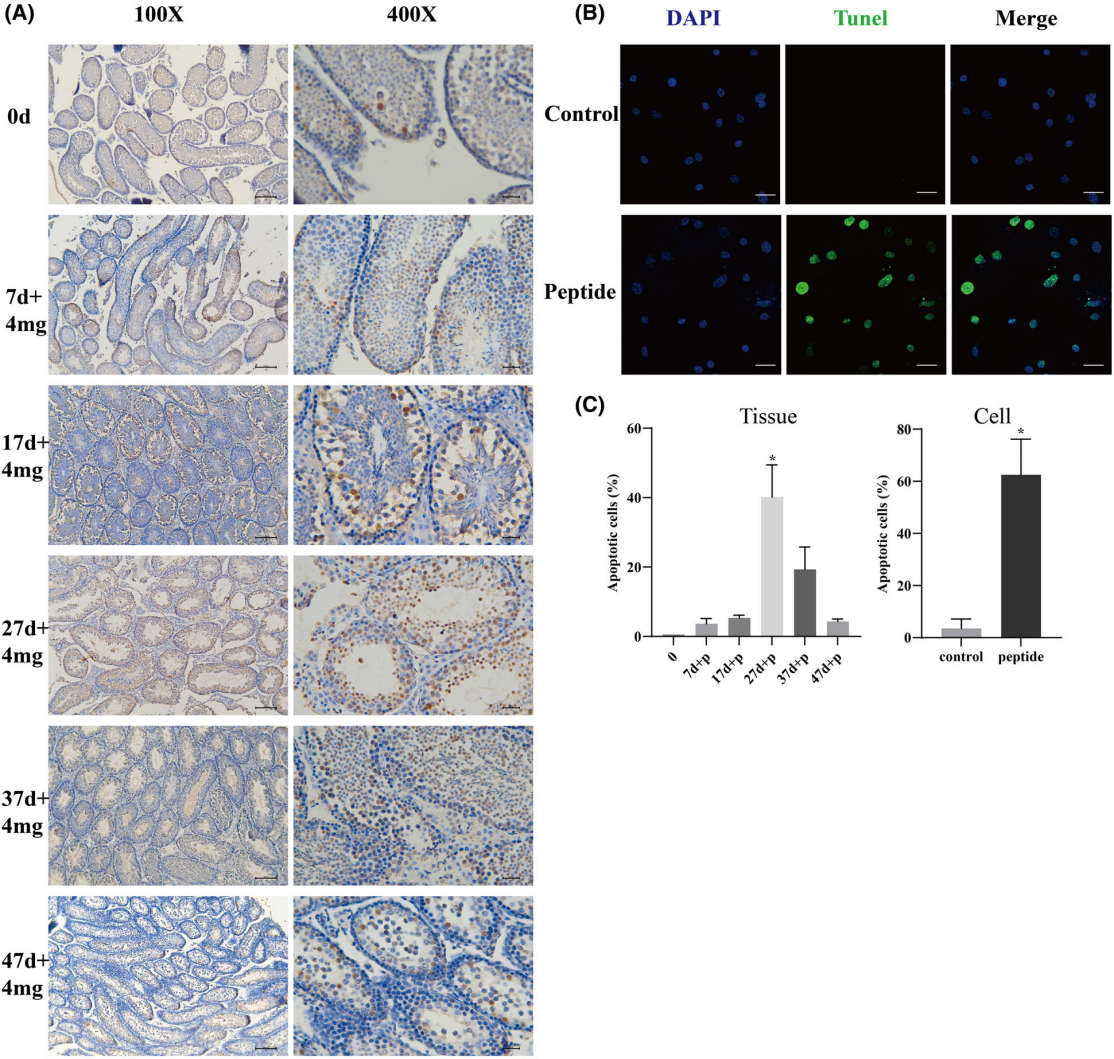
Short peptide‐induced apoptosis in testes. Testis immunohistochemistry combined with TUNEL staining to analyze apoptosis in testis (A) from six different groups and Sertoli cells (B). (C) Quantitative analysis of (A) and (B). The data represent the mean ± SEM of three independent experiments (*P < 0.05 vs. control, mean ± SEM, *n* = 3), Scale bar = 20 μm.

The peptide promotes the infiltration of immune cells, the release of inflammatory factors, and the upregulation of mitochondrial autophagy genes, all of which disrupt the TJs. We considerd whether this further affects sperm production? We used the TUNEL assay to detect cell apoptosis in the testis. As shown in Fig. 5A, TUNEL staining revealed significant apoptotic activity in Leydig cells, spermatogonia, spermatocytes and post‐meiotic cells at 27 days, with reduced activity observed at 37 and 47 days after treatment. Using immunofluorescence to observe isolated Sertoli cells allows for a clearer demonstration of the upregulation of apoptosis levels of Sertoli cells, and apoptosis of Sertoli cells directly leads to the disruption of the TJs (Fig. 5B,C). These results show that the short peptide influences the apoptosis of Sertoli cells and spermatogonia, thereby inhibiting spermatogenesis.

## Discussion

Spermatogenesis is a complex process in which germ cells develop from spermatogonia to mature spermatozoa, occurring within the seminiferous tubules of the testes [26, 27]. The BTB creates a distinct microenvironment for the development and maturation of germ cells [3]. Reproductive dysfunction may result from disruptions in BTB integrity, including environmental factors [28] and protein‐active peptides hydrolyzed by MMP family members. According to our previous studies, the 22AA peptide destroyed TJ by downregulating occludin and promoting SC apoptosis. In the present study, we further investigated the mechanism by which short peptides influence spermatogenesis.

The testicular macrophages are the primary immune cells in the testes, playing crucial roles in sperm development [29] and immunosuppression [30]. Furthermore, the immune response of testicular macrophages is lower than that of macrophages in the bloodstream. Pro‐inflammatory M1 macrophages [31] secrete high levels of pro‐inflammatory cytokines, such as TNF‐α and IL‐1β [32]. Regulatory T cells (Tregs) maintain immune tolerance in the testes, promoting sperm production and maturation [33, 34]. Under inflammatory conditions, pathogenic T cells can overwhelm the inhibitory mechanisms of Tregs by altering the balance to support autoimmune responses. Recent studies indicate that increased infiltration of CD4 T cells leads to depletion of Tregs, resulting in the breakdown of immune tolerance and causing severe immunological male subfertility [33].

Therefore, we investigated the impact of disrupting TJ on immune infiltration using short peptides. We utilized multiple immunofluorescence analyses and observed a significant increase in infiltrating CD4 T cells and macrophages in the seminiferous tubules at 17 and 27 days. However, infiltration levels began to decrease at 37 and 47 days, revealing the dynamic changes in immune cell infiltration during the process of TJ reconstruction.

Local inflammatory conditions of the testis, as well as systemic inflammation, have negative impacts on spermatogenesis for different reasons, such as bacterial infections and viral infections, which cause an invasion of monocyte‐derived macrophages, with increased production of pro‐inflammatory cytokines such as IL‐6, TNF‐α, IL‐17, IL‐1β and chemokines by resident testicular cells, resulting in a negative impact on spermatogenesis and steroidogenesis [35]. Therefore, investigating testicular inflammatory responses is extremely important, encompassing not only the infiltration of immune cells, but also the release of various signaling molecules within testicular cells. IL‐6 was significantly increased in Sertoli and extratubular cells in the seminiferous tubules of the testes, indicating the inflammatory seminiferous environment of the testes [36]. MuV infection upregulates the production of TNF‐α in mouse Sertoli cells, inducing the production of CXCL10 in an autocrine manner, which, in turn, can induce apoptosis of germ cells through paracrine signaling [37]. Recent studies on the impact of COVID‐19 on the male reproductive system suggest that, after SARS‐CoV‐2 infection, the expression of TJ proteins, including occludin and claudin‐11, is disrupted, accompanied by increased expression of pro‐inflammatory cytokines such as IL‐6, TNF‐α and IL‐1β, leading to a decrease in the number of Sertoli cells and sperm cells [38]. Thus, we focused on monitoring the dynamic changes of IL‐6 and TNF‐α during the process of peptide‐induced disruption of TJ. The expression levels of TNF‐α and IL‐6 were significantly upregulated at 7 days, reaching peak levels at 27 and 17 days, respectively, followed by a gradual decline. At 47 days, TNF‐α showed no significant difference compared to the control group at 47 days. Similarly, elevated expression levels of TNF‐α and IL‐6 were observed in Sertoli cells compared to the untreated control group. Recent research has reported that occludin expression is reduced by TNF‐α which induces apoptosis [39]. Perhaps this is a negative feedback regulation, although the trend of decreased levels of pro‐inflammatory cytokines and apoptosis at 37 and 47 days warrants further investigation.

Autophagy plays a crucial role in spermatogenesis by enabling Sertoli cells to engulf residual spermatogonia and regulating the stability of chromatoid bodies in spermatocytes [40]. In the cadmium‐induced testicular injury model in mice, mitochondrial fission proteins (DRP1 and FIS1) were increased, and mitochondrial fusion proteins (OPA1 and MFN1) were decreased, which resulted in excessive mitochondrial fission and cell apoptosis [41]. In the present study, simultaneous upregulation of mitochondrial fission protein DRP1, mitochondrial autophagy protein PINK and PARKIN protein was detected in testicular tissue and Sertoli cells at 7 days, accompanied by downregulation of mitochondrial fusion protein MFN1. Using multiple immunofluorescence techniques, it was discovered that the localization of PINK and PARKIN proteins increased in the Sertoli cells, Leydig cells and some of the spermatogenic cells of the seminiferous tubules. However, this phenomenon is not irreversible. At 37 and 47 days, the dysfunction of autophagy began to attenuate. Thus, despite our short peptide affecting the dynamic equilibrium of mitochondrial and fusion autophagy, this disruption phenomenon gradually diminished through self‐regulation.

Altough our previous study demonstrated that 22AA peptide induces occludin degradation [19], a hallmark of TJ instability, the current findings extend this observation by revealing a cascade of immune and mitochondrial perturbations. Notably, occludin loss alone may not fully recapitulate BTB dysfunction because compensatory mechanisms involving claudin‐11 and zonula occludens‐1 could transiently maintain barrier integrity [8]. Here, immune cell‐derived cytokines (TNF‐α) likely amplify Sertoli cell stress, as evidenced by mitochondrial fragmentation and mitophagy, ultimately destabilizing the spermatogenic niche. Whether TJ disruption initiates this cascade or results from secondary inflammation remains unclear. Future studies integrating claudin‐11 analysis and functional BTB permeability assays are needed to delineate the spatiotemporal relationship between TJ integrity and immune/mitochondrial dysregulation.

TNF‐α alters mitochondrial function, increases NO production and consequently reduces sperm motility [20]. Further research is needed to explore the relationship between pro‐inflammatory cytokines and mitochondrial dynamics balance during the disruption of the TJs of the BTB by the short peptide. Recent advancements in male contraception research have revealed that CDD‐2807, a specific inhibitor targeting the kinase domain of STK33, effectively reduces sperm motility and count. Importantly, these effects are reversible because sperm motility and count can be restored to normal levels after a period of discontinued use, thereby preserving fertility [42]. Our research indicates that after the administration of short peptides, the changes in immune infiltration and the dynamic balance of mitochondria require a longer period of observation to determine whether they will return to the desired levels and achieve reversibility of TJ disruption. This will provide valuable insights for the development of non‐hormonal male contraceptives.

Zhu *et al*. [43] demonstrated that elevated inflammatory cytokines (TNF‐α, IL‐1β and IL‐6) in Hadh−/− mice impair sperm motility and induce spermatocyte apoptosis, with TNF‐α inhibition effectively attenuating germ cell death, thereby underscoring the pivotal role of cytokine dysregulation in driving apoptotic processes. In the present study, the persistence of TUNEL positivity in spermatocytes and post‐meiotic cells likely stems from unresolved inflammatory microenvironments triggered by short peptide‐induced TJ disruption, in which synergistic upregulation of TNF‐α and IL‐6 propagates apoptotic signaling even during advanced spermatogenic stages. Importantly, the significant reduction in both inflammatory cytokine secretion and apoptosis levels by 47 days further corroborates the existence of a cytokine‐driven apoptotic cascade, highlighting its time‐dependent attenuation following initial insult.

In summary, the present study elucidates how 22AA peptide impairs spermatogenesis through immune dysregulation and mitochondrial dysfunction. Although occludin degradation [19] may initiate BTB perturbation, the sustained inflammatory microenvironment and mitophagy likely drive germ cell apoptosis. These findings highlight the peptide's potential as a contraceptive agent targeting the testicular niche, although future studies must clarify the causal relationship between TJ integrity and immune/mitochondrial pathways.

## Conflicts of interest

The authors declare that they have no conflicts of interest.

## Peer review

The peer review history for this article is available at https://www.webofscience.com/api/gateway/wos/peer‐review/10.1002/2211‐5463.70058.

## Author contributions

HW and DC conceived and designed the project. HW, XT and DC acquired the data. HW and XT analyzed and interpreted the data. HW and DC wrote the paper.

## Supporting information


**Fig. S1.** Multiple immunofluorescence assays were used to detect infiltration of immune cells.
**Fig. S2.** Multiple immunofluorescence assays were used to detect Parkin expression and localization.
**Fig. S3.** Multiple immunofluorescence assays were used to detect Pink expression and localization.

## Data Availability

The data that support the findings of this study are available from the corresponding author upon reasonable request.
