# Supplementary material for: Short peptide perturbs spermatogenesis via immune microenvironment dysregulation and mitochondrial imbalance
Source: FEBS Open Bio. 2025 May 22;15(9):1485–95. doi: 10.1002/2211-5463.70058 (PMC12401178; doi:10.1002/2211-5463.70058)

**Fig. S1** Multiple immunofluorescence assays were used to detect infiltration of immune cells. Scale bar: 20 μm.
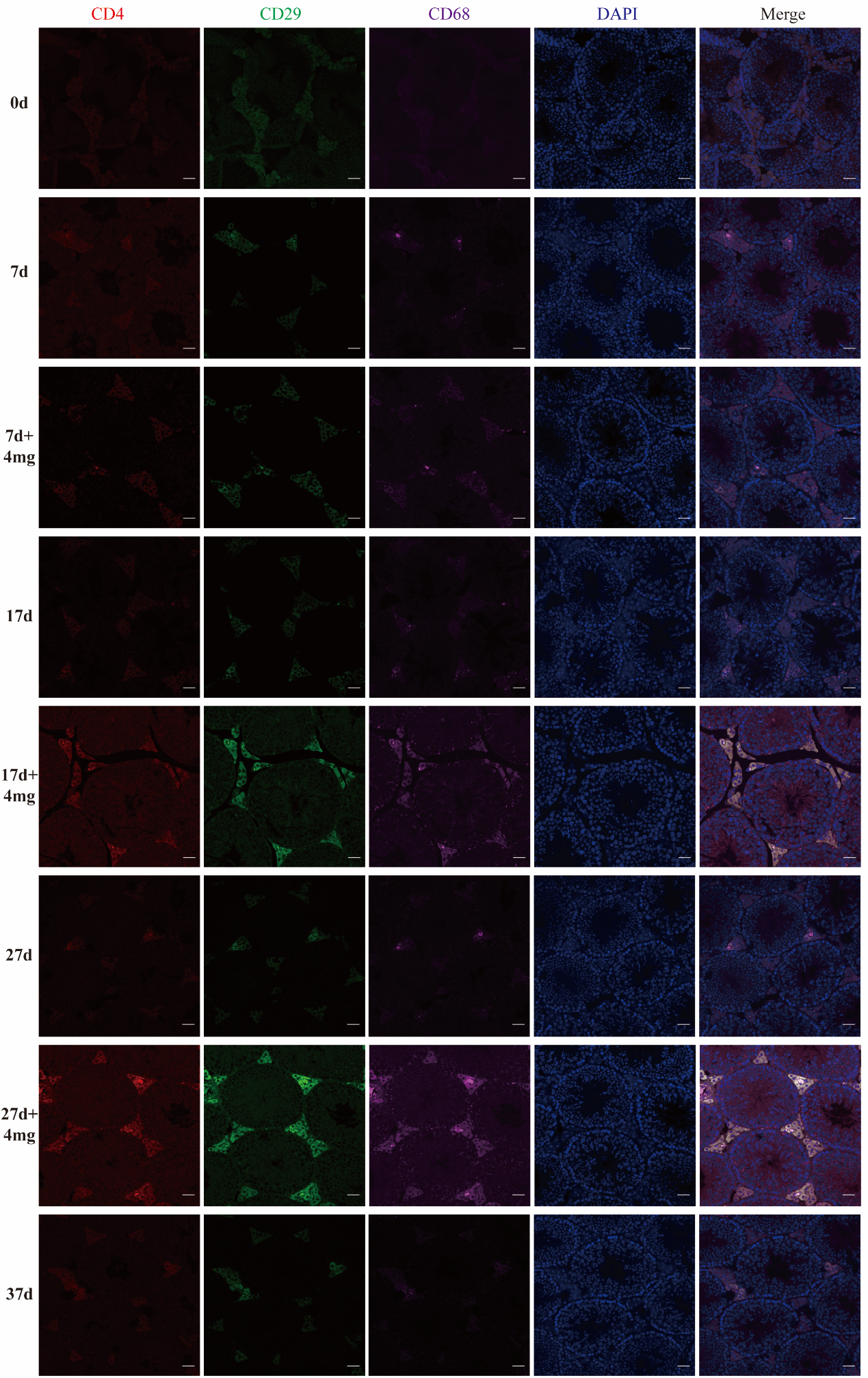


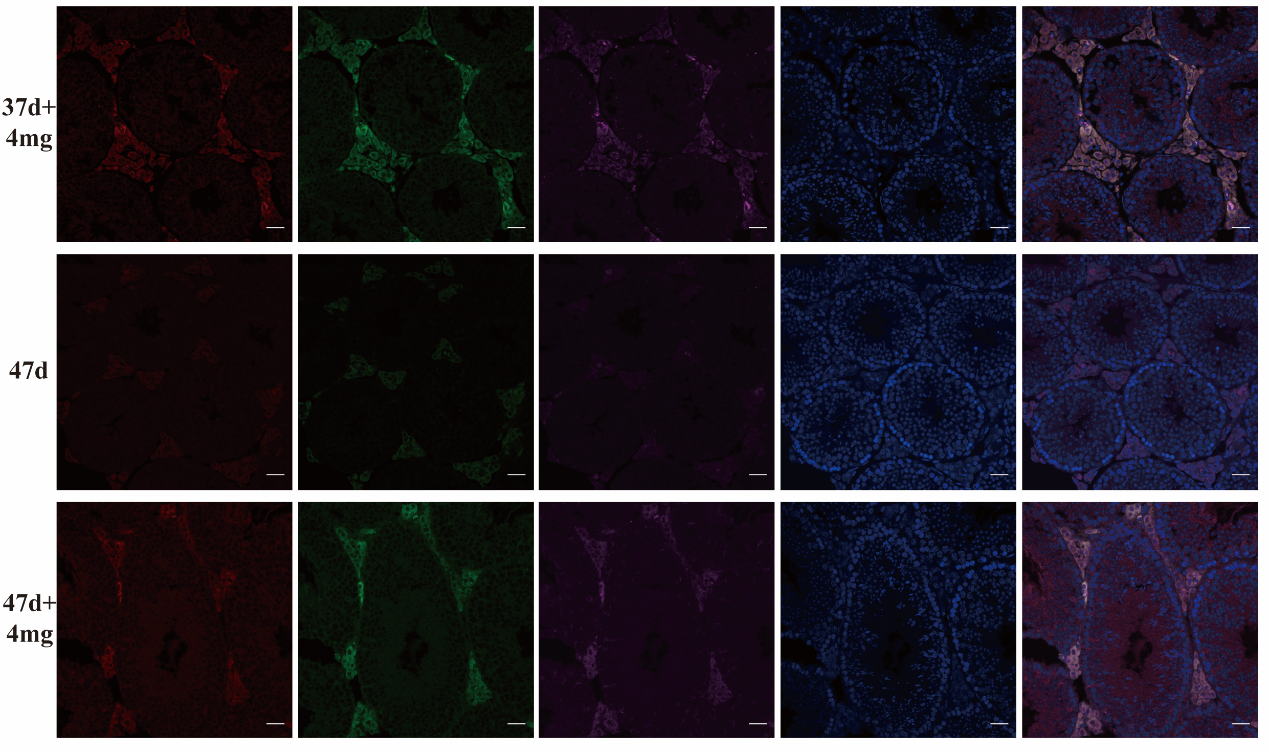


**Fig. S2** Multiple immunofluorescence assays were used to detect Parkin expression and localization. Scale bar: 20 μm.
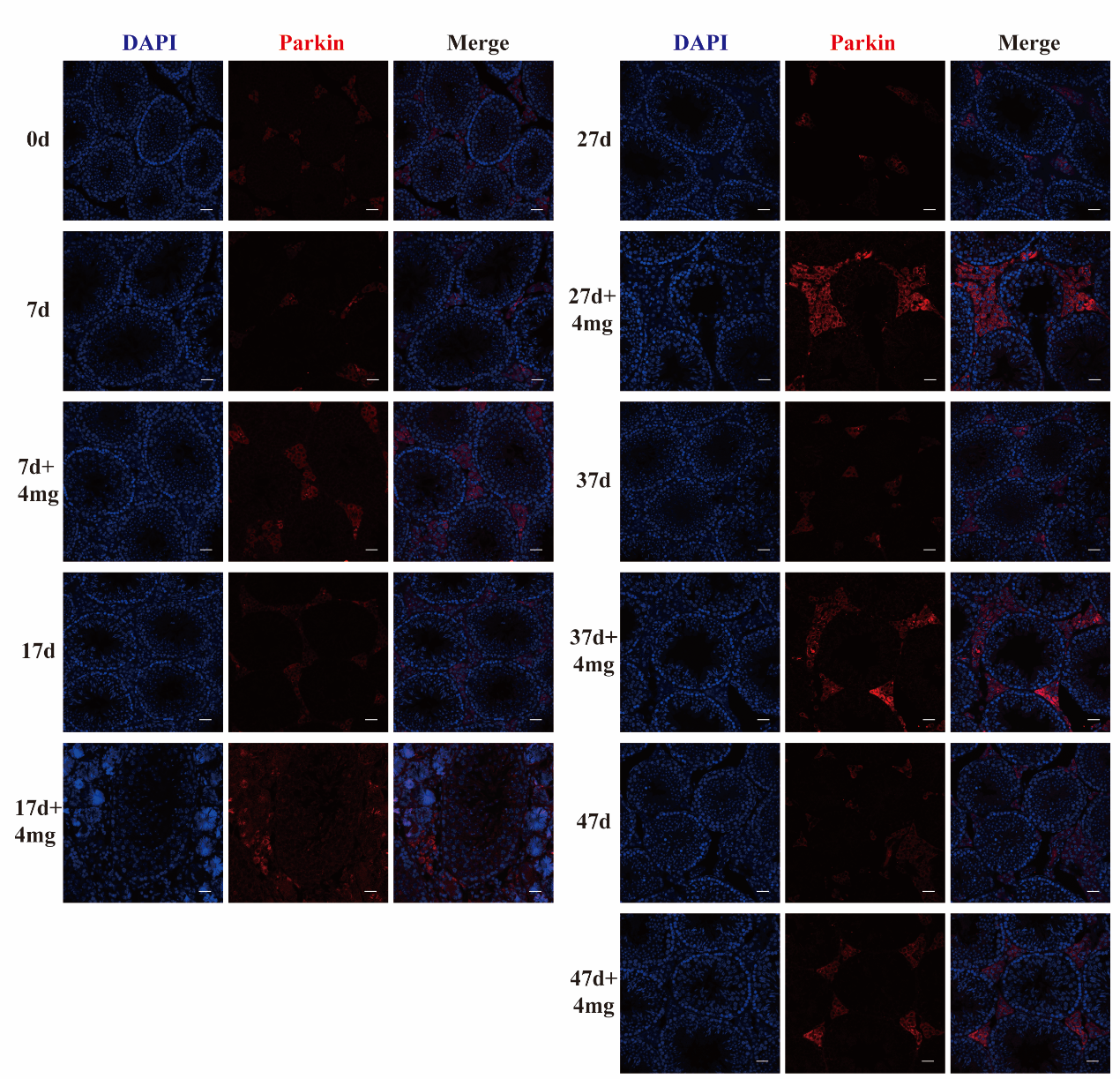


**Fig. S3** Multiple immunofluorescence assays were used to detect Pink expression and localization. Scale bar: 20 μm.
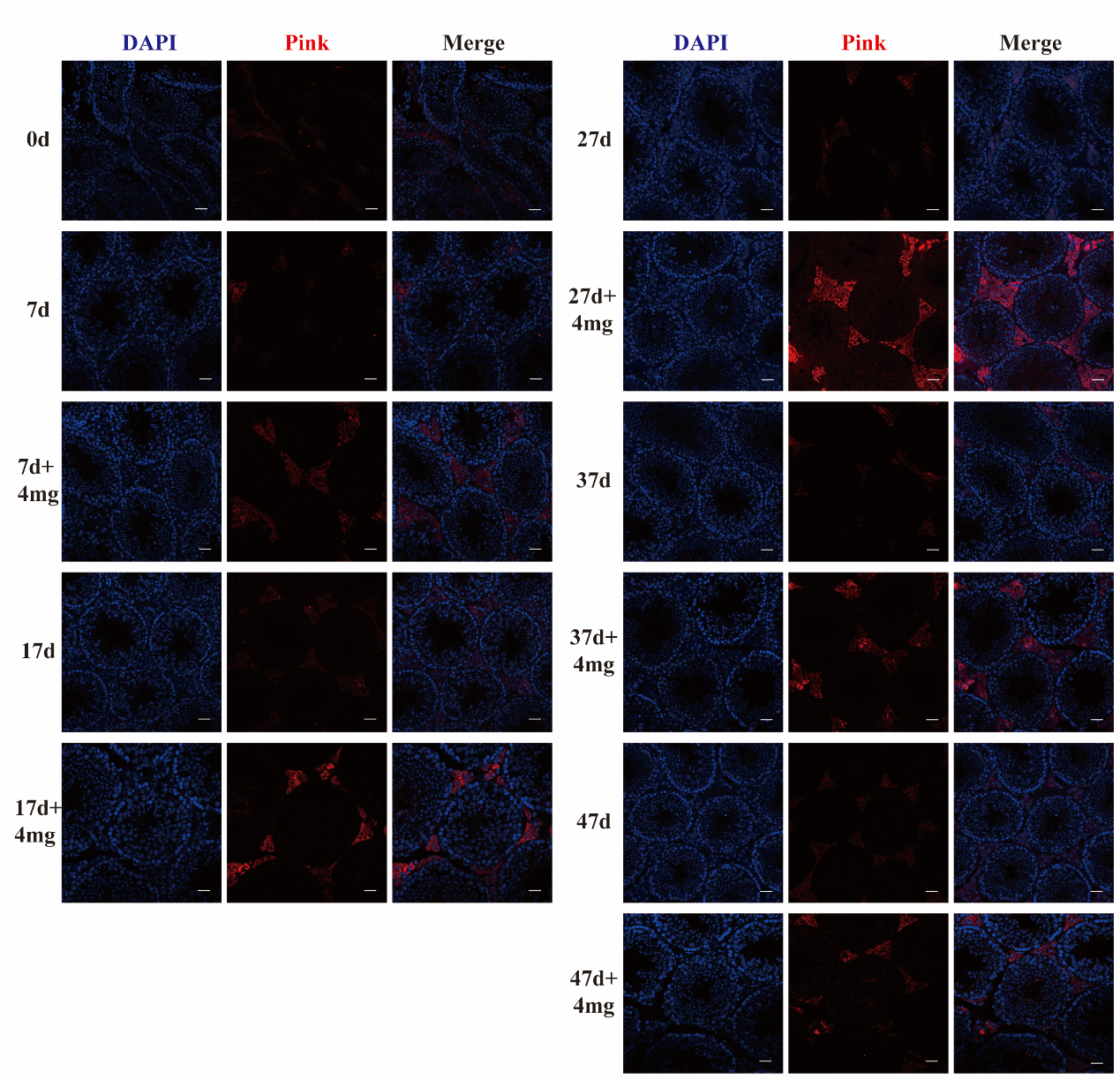

Supplement: Supplementary file 1 — Fig. S1. Multiple immunofluorescence assays were used to detect infiltration of immune cells. Fig. S2. Multiple immunofluorescence assays were used to detect Parkin expression and localization. Fig. S3. Multiple immunofluorescence assays were used to detect Pink expression and localization. [file FEB4-15-1485-s001.docx]
